# The Testosterone Test: Phthalate Inhibits Leydig Cell Aggregation

**Published:** 2007-03

**Authors:** Julia R. Barrett

Testicular cancer and low sperm count are adult disorders, but evidence increasingly suggests they have a fetal origin. Cryptorchidism and hypospadias, apparent at birth, also appear linked to prebirth events. According to the testicular dysgenesis syndrome (TDS) hypothesis, all four disorders, which by some reports have become more common in recent decades, partially stem from fetal abnormalities in testosterone-producing Leydig cells. An investigation now reveals that di(*n*-butyl) phthalate (DBP) and its metabolite monobutyl phthalate (MBP) suppress testosterone production in rats and primates **[*EHP* 115:390–396; Hallmark et al.]**. Attempts to establish *in vitro* models were unsuccessful, however.

In rats, prenatal exposure to DBP can induce Leydig cell changes and TDS-like effects. Chronic, low-level exposure to DBP and other phthalates, widely used as plasticizers, is common among humans, but it is unknown if it causes the same effects. The primary goal of the current study was to determine whether effects seen in rats could be replicated *in vitro* with fetal rat and human testis explants (extracted tissue maintained in culture). The team also conducted experiments in male infant marmosets, whose neonatal testosterone production mirrors that of human males.

Preliminary work revealed that rats with prenatal DBP exposure produced significantly less testosterone and had more medium or large Leydig cell clusters. This is notable because larger clusters are associated with defective testicular development. Rat fetal testis explants, however, showed only minor MBP-related effects, and results from comparable human explants were even less conclusive.

Because known *in vivo* reactions could not be replicated *in vitro*—indicating either a problem with the method or misidentification of the active metabolite—the team tested MBP in marmosets. In five sets of marmoset twins, one twin was exposed to MBP for two weeks while the other served as a control. Blood testosterone levels did not differ significantly, but Leydig cell numbers and size were consistently increased in the MBP group.

Because low testosterone triggers increased secretion of luteinizing hormone, which stimulates Leydig cell testosterone production, the researchers checked whether there was an initial MBP-associated suppression in testosterone production. They found that a single dose of MBP in newborn marmosets significantly reduced testosterone levels within hours. This finding led to the hypothesis that increased luteinizing hormone secretion compensates for an initial MBP-associated inhibition of testosterone production, which the researchers conclude should be considered in future animal studies. They also conclude that *in vivo* marmoset research represents the best current means for investigating the steroidogenic effects of DBP relevant to humans.

## Figures and Tables

**Figure:**
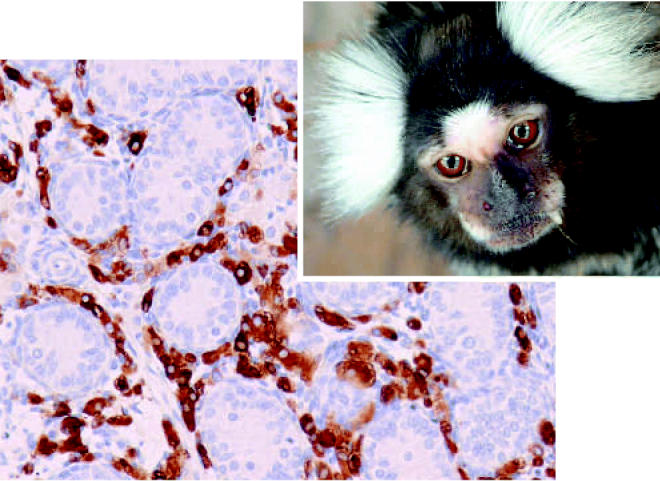
Testicular effect The numbers and size of Leydig cells (in brown, above) increased in MBP-treated marmosets.

